# Preparation of Nanocomposite-Based High Performance Organic Field Effect Transistor via Solution Floating Method and Mechanical Property Evaluation

**DOI:** 10.3390/polym12051046

**Published:** 2020-05-02

**Authors:** Youn Kim, Yeon Ju Kwon, Seungwan Ryu, Cheol Jin Lee, Jea Uk Lee

**Affiliations:** 1 Carbon Frontier Research Center, Korea Research Institute of Chemical Technology (KRICT), Daejeon 34114, Korea; younkim@krict.re.kr (Y.K.); kyj0905@krict.re.kr (Y.J.K.); skyzoop@krict.re.kr (S.R.); 2 School of Electrical engineering, Korea University, Seoul 02841, Korea

**Keywords:** organic field-effect transistor, nanocomposites, electrochemically exfoliated graphene, solution floating method, film-on-elastomer

## Abstract

We demonstrate that using nanocomposite thin films consisting of semiconducting polymer, poly(3-hexylthiophene) (P3HT), and electrochemically exfoliated graphene (EEG) for the active channel layer of organic field-effect transistors (OFETs) improves both device performances and mechanical properties. The nanocomposite film was developed by directly blending P3HT solution with a dispersion of EEG at various weight proportions and simply transferring to an Si/SiO2 substrate by the solution floating method. The OFET based on P3HT/EEG nanocomposite film showed approximately twice higher field-effect mobility of 0.0391 cm^2^·V^−1^·s^−1^ and one order of magnitude greater on/off ratio of ~10^4^ compared with the OFET based on pristine P3HT. We also measured the mechanical properties of P3HT/EEG nanocomposite film via film-on-elastomer methods, which confirms that the P3HT/EEG nanocomposite film exhibited approximately 2.4 times higher modulus (3.29 GPa) than that of the P3HT film (1.38 GPa), while maintaining the good bending flexibility and durability over 10.0% of bending strain and bending cycles (1000 cycles). It was proved that the polymer hybridization technique, which involves adding EEG to a conjugated polymer, is a powerful route for enhancing both device performances and mechanical properties while maintaining the flexible characteristics of OFET devices.

## 1. Introduction

Organic semiconductors have several advantages over inorganic semiconductors such as flexibility, solution processability, mass production, and low-cost manufacturing, which allow the fabrication of a wide range of electronic devices [1]. Among organic semiconductors, conjugated polymer semiconductors such as poly(3-alkylthiophene) (P3AT) [2,3], poly(3,3‴-didodecylquaterthiophene) (PQT-12) [4,5], and diketopyrrolopyrrole (DPP)-based copolymer [6,7] have been synthesized for use as active layers of organic electronic devices, such as organic solar cells (OSCs) [8,9], organic field-effect transistors (OFETs) [10], organic light-emitting diodes (OLEDs) [11], and sensors [12]. However, current conjugated polymer semiconductors still have some limitations; they have low carrier mobilities (0.001–0.01 cm^2^·V^−1^·s^−1^), as well as poor uniformity and stability when they are deposited to fabricate organic devices [13,14]. To industrialize the organic electronic devices, the mobility of conjugated polymer semiconductors needs to be increased while maintaining its unique flexibility and solution-processability.

There are two strategies for improving the mobility of a conjugated polymer semiconductor. The first involves synthetic methods that directly change the length of the backbone, conjugate moieties, and regioregularities in the conjugated polymer. However, these synthetic methods follow complex routes and need long times to synthesize the required products [7,15,16]. The second strategy involves the hybridization of conjugated polymer and nanocarbon materials such as carbon nanotubes (CNT) and graphenes, since the nanocarbons have excellent intrinsic electrical and mechanical properties (charge carrier mobility of 200,000 cm^2^ V^−1^ s^−1^, electrical conductivity of 6000 S cm^−1^, and Young’s modulus of 1100 TPa) [17,18,19]. Geng et al. reported a remarkable enhancement in the field-effect mobility by blending single-walled carbon nanotubes (SWCNTs) into a semiconductor polythiophene film [20]. Huang et al. showed that the field-effect mobility of a semiconducting polymer/graphene hybrid OFET was four times higher than that of OFETs based on pure semiconducting polymers [21]. These studies have discussed that the inclusion of functional nanocarbons can improve the morphology and increase the crystallinity of the active channel layers of the organic electronic devices. Furthermore, the nanocarbons can act as conducting bridges between the crystalline regions of the semiconductor film and provide “fast lanes” for charge carriers, thereby enhancing the effective mobility of the entire transistor [22,23].

In this research, we fabricated an OFET based on nanocomposite films of conjugated polymer semiconductors as the matrix and electrochemically exfoliated graphene (EEG) as fillers using the solution-floating method. The exfoliated graphene was mass-produced via an electrochemical method described in detail in our previous reports [24,25]. We used poly(3-hexylthiophene) (P3HT), one of the most commonly used conjugated polymer semiconductor for active channel layer of organic solar cell and transistor devices. The P3HT solution in chloroform was simply mixed with EEG dispersed in *N*-methyl-2-pyrrolidone (NMP) to prepare a hybrid solution, hereon referred to as P3HT/EEG. The pristine P3HT and P3HT/EEG nanocomposite are solution-processable and allow several deposition methods such as spin coating, inkjet printing, and roll-to-roll method. In this work, we fabricated the OFET via the solution-floating method [26], which is a simple and convenient method to provide high-quality crystalline films and to prepare active layers made of hybrid materials [27,28,29]. The OFET based on P3HT/EEG nanocomposite film showed approximately twice higher field-effect mobility of 0.0391 cm^2^·V^−1^·s^−1^ and one order of magnitude greater on/off ratio of ~10^4^ compared with the OFET based on pristine P3HT.

Next, we examined the mechanical properties of P3HT/EEG nanocomposite films using film-on-elastomer (FOE) methods including buckling and bending tests. We found that the P3HT/EEG nanocomposite film exhibited approximately 2.4 times higher modulus (3.29 GPa) than that of the P3HT film (1.38 GPa), while maintaining the good bending flexibility and durability over 10.0% of bending strain (bending radius of 1.5 cm) and bending cycles (1000 cycles). The enhanced mechanical properties of the nanocomposite films are attributed to the enhancement of the film roughness and crystallinity, where the P3HT and EEG are tightly held together compared to an amorphous structure.

## 2. Materials and Methods

### 2.1. Materials

Graphite foils were purchased from Alfa Aesar (99.8 %, Haverhill, MA, United States). Ammonium sulfate ((NH_4_)_2_SO_4_, 99.5%) and *N*-methyl-2-pyrrolidone (NMP, 99.5%, special grade) were purchased from Samchun Chemical Company (Seoul, Korea). Poly(3-hexylthiophene) (MW = 50–70 KDa, PDI = 1.4–1.6) was purchased from Solaris Chem Inc (Vaudreuil-Dorion, QC, Canada). Toluene (anhydrous, ≥99%) was purchased from Sigma-Aldrich (Milwaukee, WI, USA). The membrane filter (Anodisc 47) was purchased from Whatman (Maidstone, UK). Poly(dimethylsiloxane) (PDMS, Sylgard 184) was purchased from Dow Corning (Midland, MI, USA).

### 2.2. Solution-Floating Method for Fabrication of P3HT/EEG Nanocomposite Films

P3HT solutions were dissolved in toluene at a concentration of 10.0 mg mL^−1^ and filtered using a syringe filter with 0.2 μm pores. EEG was dispersed in NMP at concentrations of 0.25, 0.5, 1.0, 2.0 mg mL^−1^ by ultrasonication for 12 h. Subsequently, 1 mL of pristine P3HT solution was added into 1 mL EEG dispersion (P3HT solution:EEG dispersion = 1:1 (*v*/*v*)) and sonicated for 60 min. The mass ratios of P3HT:EEG in the resulting four different nanocomposite solutions were 100:2.5, 100:5, 100:10, and 100:20, and the solutions were named as P3HT/EEG_2.5_, P3HT/EEG_5_, P3HT/EEG_10_, and P3HT/EEG_20_ nanocomposite solution, respectively, where the subscripts represented a “parts per hundred resin (phr)”. For each solution, 30 μL was dropped onto water in a petri dish (diameter: 100 mm) and maintained at room temperature until the completion of solvent evaporation.

### 2.3. Fabrication of OFETs based on P3HT/EEG Nanocomposite Films

A highly p-doped Si wafer with a thermally grown SiO_2_ layer (thickness: 300 nm) was cleaned with distilled water, ethanol, and acetone for 30 min each using an ultrasonicator. The nanocomposite films were formed by the solution floating method. The nanocomposite films were transferred onto the cleaned Si wafer by gently dropping the Si substrates into the nanocomposite film floated on water. After transfer, annealing was carried out at 120 °C for 10 min. The Au electrodes (thickness: 100 nm) were thermally evaporated through a shadow mask (channel length: 50 μm; channel width: 2000 μm). The field-effect mobility (*μ*_FET_) was calculated in the linear regime using the standard FET equation. A pristine P3HT thin film device was also prepared for comparison by the solution-floating method with P3HT toluene solution added to the same volume of pure NMP (P3HT solution:NMP = 1:1 (*v*/*v*)).

### 2.4. Measurements of Mechanical Properties of Nanocomposite Films (FOE Method)

Buckling method (tensile modulus): Liquid PDMS (Sylgard 184, base:cross-linker = 10:1) was poured into a petri dish and cured at 50 °C for 2 h in a vacuum oven. The cured PDMS was cut into a rectangular shape (length = 60 mm, width = 10 mm, height = 3 mm) and was fixed on both ends onto a glass slide with 10% strain [30,31]. The pre-strained PDMS was treated with UV-ozone (UV-O_3_) and P3HT and P3HT/EEG nanocomposite films made by solution-floating method were deposited on the surface. After drying at room temperature, the pre-strained PDMS was released to its initial state.

Bending strain: The P3HT and P3HT/EEG nanocomposite films were deposited on the UV-O_3_ treated surfaces of PDMS rectangles, which were fixed onto glass slides. The samples were set on a translation stage for the bending process. The initial length (*L*) of the samples was changed by compression forces on the stage. The samples were bent in the vertical (upper) direction, and the length of the samples was decreased from *L* to *L-dL* by bending. The bending radius (*R*_bend_) and strain (ε) were calculated using *dL/L* [28]. SEM images of the film surfaces were acquired at fixed *dL/L* = 50% and 80% (see Figure S1, Supporting Information).

Cyclic bending test: Poly(3,4-ethylenedioxythiophene)-poly(styrenesulfonate) (PEDOT:PSS) (PH 1000) aqueous solution with 1 wt% sodium dodecyl sulfate (SDS) was filtered using a PTFE syringe filter with 1.0 μm pore size and spin-coated onto the PDMS substrate at a spin speed of 500 rpm for 120 s followed by 2000 rpm for 30 s. The PDMS/PEDOT:PSS substrate was dried for 12 h at the room temperature. After that, the P3HT/EEG nanocomposite films were transferred onto the PDMS/PEDOT:PSS substrate by using solution floating method. The cyclic bending test was performed at *dL/L* = 0 to 50% for 1000 cycles. At 200, 400, 600, 800, and 1000 cycles, we took off the P3HT/EEG nanocomposite films from the PDMS/PEDOT:PSS substrate; at each bending cycle, P3HT/EEG nanocomposite film on the PDMS/PEDOT:PSS substrate was immersed and floated on water surface because the PEDOT:PSS layer was dissolved by water penetration. The floated nanocomposite film was transferred onto the Si/SiO_2_ substrate with Au source/drain (S/D) electrodes for measuring the field-effect mobility.

### 2.5. Characterization

The output and transfer characteristics of the OFET devices were measured at ambient condition using a Keithley 2612B source meter (Cleveland, OH, USA) and an MST-4000A MSTECH probe station (Hwaseong, Korea). The crystallinity of the nanocomposite films was determined by various spectroscopic analyses. UV–Vis spectra were collected using an Optizen POP (K Lab Co., Daejeon, Korea). Raman spectra were recorded with a Rigaku Ultima IV (laser wavelength: 532 nm, Kyoto, Japan). The X-ray diffraction (XRD) patterns of the nanocomposite films were acquired using a Rigaku Ultima IV diffractometer (Kyoto, Japan). The surface morphology and topographies of the nanocomposite films were investigated using scanning electron microscopy (SEM) (COXEM, CX-200TA, Daejeon, Korea) and atomic force microscopy (AFM) (Bruker, Nanoscope, Billerica, MA, USA).

## 3. Results and Discussion

### 3.1. Preparation and Characterization of OFET based on the P3HT/EEG Nanocomposite Films via Solution Floating Method

**Figure 1:**
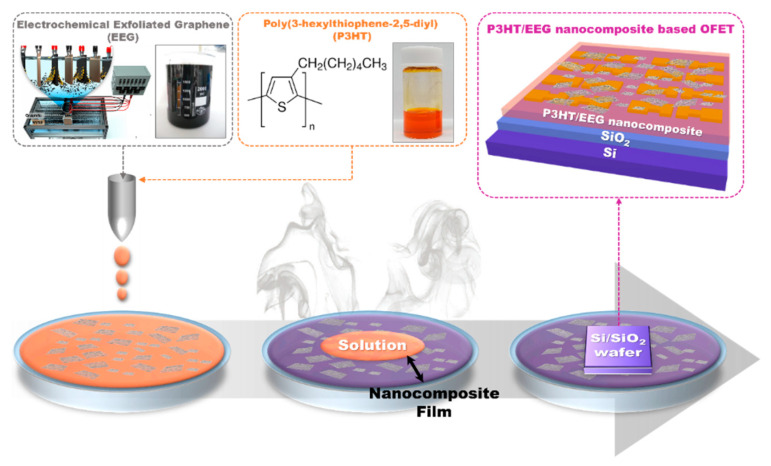
Schematic illustration of fabrication process of poly(3-hexylthiophene) (P3HT)/electrochemically exfoliated graphene (EEG) nanocomposite film and organic field-effect transistor (OFET) device via solution floating method.

The OFET based on the P3HT/EEG nanocomposites was fabricated via the solution floating method on a Si/SiO_2_ substrate (Figure 1). The nanocomposite materials for the active channel layer of the OFET devices were dissolved in toluene/NMP mixed solvent and the solution was dropped onto water in a petri dish. The toluene/NMP is a suitable solvent for preparing uniform nanocomposite films via solution floating method due to the following reasons: First, the NMP is miscible with water and several common organic solvents. When P3HT/EEG nanocomposite, dissolved in toluene/NMP mixed solvent, was dropped onto water surface, the NMP solvent with high boiling point was diffused into the water and the toluene solvent was evaporated from the water surface. As a result, the NMP and toluene solvents were easily removed and the P3HT/EEG nanocomposite thin film remained on the water surface. The thin film can be easily transferred by stamping onto the SiO_2_ dielectric layer to form the active layer of the OFET device. Second, it has been reported that the OFET device fabricated by solution floating method with P3HT channel layer using toluene solvent showed better electrical properties than those of devices prepared using chloroform and tetrahydrofuran (THF) solvents [27]. A floated thin film composed of well-organized P3HT nanowires can be obtained by using a water immiscible toluene solvent with a slow evaporation rate, while the chloroform and THF solvents induced random aggregation of P3HT molecules instead of well-organized P3HT nanowires because of their fast evaporation rate. Consequently, when the toluene/NMP mixed solvent was used for the solution-floating method, the crystallinity of P3HT/EEG nanocomposite thin film increased and the OFET devices based on the nanocomposite channel layers showed the enhanced field-effect mobilities.

**Figure 2:**
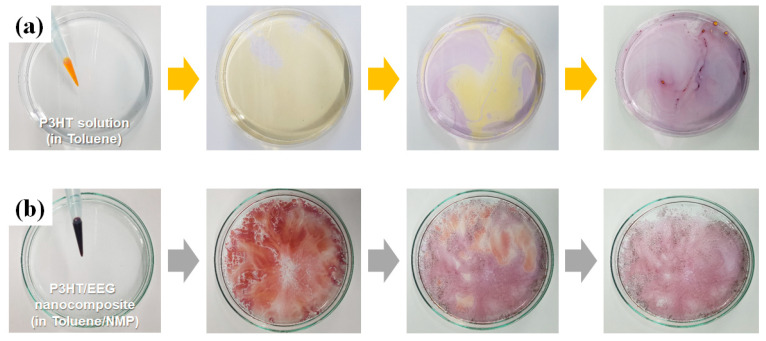
Digital images of fabrication process of (**a**) pristine P3HT and (**b**) P3HT/EEG nanocomposite films via solution floating method.

Figure 2 shows the thin film fabrication process of pristine P3HT and P3HT/EEG nanocomposite via solution floating method. The color of the pristine P3HT solution changed from orange to purple when the toluene solvent evaporated, which means that the floated P3HT molecules on water surface could be self-assembled into nanowire structures via interchain π–π interactions [27]. On the other hand, the color of the P3HT/EEG nanocomposite solution was immediately changed from orange to dark purple right after the P3HT in toluene solution and EEG in NMP solution were mixed. P3HT molecules were aggregated to form self-assembled nanowire because the NMP is not a good solvent for P3HT solubilization. Oh et al. reported that the nanorods or nanofibrils of the P3HT were formed via rapid cooling of the solution, which caused the solubility changes of the solvent and gave the driving force for one-dimensional (1D) growth between rigid conjugated backbones of P3HT [3,32]. They used P3HT nanofibrils for active layer of the solar cell and showed the increase of charge carrier mobility of the devices. For the same reason, we also made the nanocomposite solution with the P3HT nanowire and the EEG sheet using the difference in solubility between the toluene and NMP solvents. Both P3HT nanowire and the EEG sheet in the nanocomposite solution could act as seed crystals during the solution floating process, therefore the P3HT/EEG nanocomposite thin film showed a better crystallinity than that of the pristine P3HT film and had a well-organized morphology [26]. The detailed characterization and explanation will be given in Figures 4 and 5.

**Figure 3:**
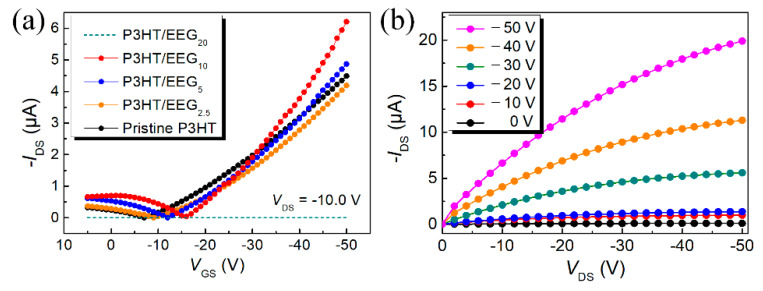
(**a**) Transfer characteristics of OFETs based on pristine P3HT and P3HT/EEG nanocomposite films. (**b**) Output characteristics of OFET based on P3HT/EEG_10_ nanocomposite film.

The OFET devices were fabricated with five different mass ratios of EEG in the solution. Figure 3a,b show the electrical (transfer and output curves) characteristics of the OFETs based on the pristine P3HT and the P3HT/EEG nanocomposite channel layers. The field-effect mobility of the devices in the linear regime was calculated using the following equation:

$$I_{\mathrm{DS}}=\frac{W}{L}\times C_{i}\times \mu_{\mathrm{FET}}\times V_{\mathrm{DS}}\times \left(V_{\mathrm{GS}}-V_{\mathrm{Th}}\right)$$

where *W* is the channel width, *L* is the channel length (*W/L* = 2000 μm/50 μm), *C*_i_ is the gate dielectric capacitance per unit area (*C*_i_ of 300 nm thick SiO_2_ ≈ 11.5 nF cm^−2^), *μ*_FET_ is the field-effect mobility, and *V*_Th_ is the threshold voltage. The P3HT/EEG_2.5_ sample had nearly similar electrical properties as the pristine sample, while the P3HT/EEG_5_ sample had slightly increased mobility (Figure 3a and Table 1). In the case of the OFET based on the P3HT/EEG_10_ nanocomposite film, the field-effect mobility (*μ*_FET_ = 0.0391 cm^2^∙V^−1^∙s^−1^) was almost double that of the pristine sample (*μ*_FET_ = 0.0223 cm^2^∙V^−1^∙s^−1^) and the on/off ratio increased by one order of magnitude. Moreover, the OFET based on the P3HT/EEG_10_ film had well-defined gate modulation in the output curves, signifying that the film has good semiconducting properties (Figure 3b). This means that there is an optimal ratio of P3HT and EEG to enhance the performance of OFETs.

Previous research has shown that hybridization of two-dimensional (2D) nano-carbon materials such as graphene and graphene oxide (GO) with organic semiconductors amplifies the electrical performance of OFETs. We have already explained that 2D graphene can increase the crystallinity of nanocomposite film. In addition, Huang et al. demonstrated that graphene sheet has outstanding electrical properties, so it can act as a bridge for connecting the crystalline regions of the P3HT grain [21]. Theoretically, in the nanocomposite system, the increases of the graphene content (covering area) can improve the electrical properties of OFET until reaching the percolation threshold of the graphene. As a result, increasing the covering area with highly conductive and large-area graphene sheets in the channel layer can enhance the electrical characteristic of the OFET such as mobility and on/off ratio than the OFET based on pristine P3HT channel layer. Therefore, increasing the mass ratios of EEG in nanocomposite films (P3HT/EEG_10_) enhanced the field-effect mobility and the on/off current ratio.

The P3HT/EEG_20_ sample with the highest content of EEG, however, did not work well as the active channel layer of the OFET devices because of the aggregation of EEG in the nanocomposite solution (Figure S2, Supporting Information). Large amounts of EEG can be dispersed well in solvents such as NMP or DMF, but not in solvents such as toluene and toluene/NMP mixed solvent; hence, aggregation can occur when the ratio of EEG is increased in the nanocomposite solution [33,34]. Therefore, in the toluene/NMP solvent system, we assumed that the 10 phr of EEG was the maximum ratio which can stably disperse the EEG in the nanocomposite solution. When the ratio of EEG reached 20 phr, the EEG was quite aggregated and separated from of the P3HT/EEG_20_ solution even after ultrasonication treatment. Furthermore, when the content of the graphene is over the optimal level (10 phr) in the nanocomposite, the crystallinities of the film and the device performances deteriorated because the structural order of P3HT decreased due to the excessive electrostatic interaction between 2D graphene sheets and P3HT molecules [21,35,36]. Therefore, the P3HT/EEG_20_ nanocomposite solution did not exhibit the electrical properties of an OFET even when a thin film was formed by the solution floating method.

**Figure 4:**
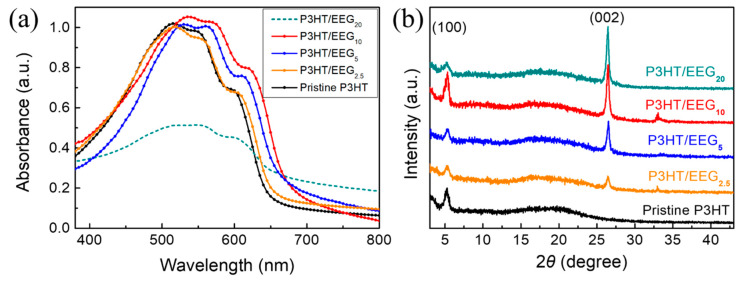
(**a**) UV–Vis spectra and (**b**) XRD patterns of transferred P3HT and P3HT/EEG nanocomposite films via solution-floating method.

The transferred P3HT thin film via the solution-floating method has strong UV–Vis absorption peaks at 515, 550, and 600 nm due to the crystalline intermolecular structure of thiophene chains (Figure 4a) [26]. The shoulder peak at 600 nm is caused by the interchain π-π* transition of P3HT chains [37]. As the EEG content increased from 0 to 10 phr, the intensity of this shoulder peak increased and the peak was red-shifted. This is due to the increase in chain motion and crystalline ordering between the *π-π* interacting 2D-EEG surface and thiophene chains of P3HT. In the case of thin film which consists of homogeneous polymer such as poly(3-alkylthiphene), the main factor of the changes in the UV–Vis spectrum is conjugate length of poly(3-alkylthiphene) [38]. The absorption peak of the P3HT thin film was red-shifted when increasing the molecular weight because the longer conjugation length of P3HT leads to absorption at lower energy. On the other hand, the P3HT/EEG nanocomposite film is heterogeneous film so that the intermolecular interaction between P3HT molecules and EEG sheets is the main factor of the change in the UV–Vis absorption peaks. The results of peak broadening and red-shift of the UV–Vis absorption of P3HT/EEG nanocomposite film demonstrate charge transfer interaction between the two materials. Pandey et al. also reported that the UV–Vis absorption peak would red-shift caused by the molecular interactions between conjugate polymer and graphene [39]. Therefore, it can be observed that the UV–Vis absorbance spectra of the P3HT/EEG nanocomposite films was red-shifted compared to that of pristine P3HT film. Meanwhile, the P3HT/EEG_20_ nanocomposite film exhibited weak absorption because of the aggregation of EEG.

XRD analysis was carried out to investigate the change of the crystallinity of the nanocomposite films (Figure 4b). The XRD profiles of pristine P3HT and P3HT/EEG nanocomposite films with different contents of EEG equally showed a sharp peak at 2θ = 5.3° due to the (100) plane of the P3HT chain. A strong diffraction peak at 26.4° is corresponding to an interlayer d-spacing of EEG sheets (3.37 Å) in the nanocomposite. Moreover, the intensities of the (100) and (002) peaks drastically increased in the P3HT/EEG_10_ nanocomposite film, which implies that the optimal content of EEG in the composite film can induce higher crystallinity and stronger intermolecular interactions. It has been reported that the P3HT molecules has more ability to crystallize in the composite film because the thiophene rings in a P3HT chain becomes interconnected with sp^2^-hybridized graphene surface [40,41].

**Figure 5:**
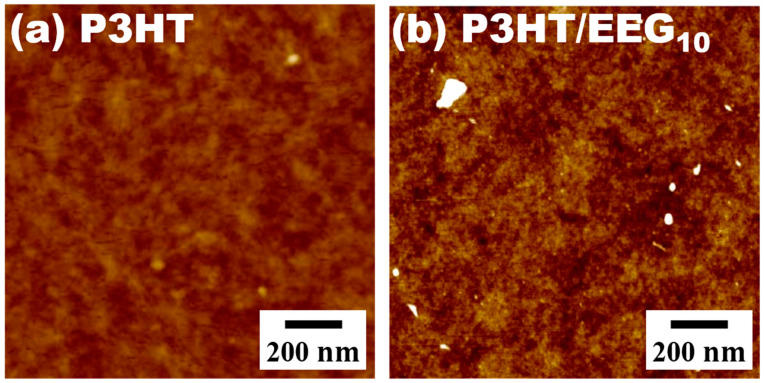
AFM topographies of transferred P3HT and P3HT/EEG_10_ nanocomposite films via solution floating method.

The AFM images of the transferred P3HT and P3HT/EEG_10_ nanocomposite films via solution floating method showed a clear topological difference (Figure 5). The P3HT/EEG_10_ nanocomposite film has more distinct worm-like structures with a higher root mean square (RMS) roughness of 2.45 nm than that of the P3HT film (RMS roughness = 1.91 nm) because of the inclusion of large crystalline domains of P3HT derived by EEG [39,42]. This also confirms that the structure of P3HT/EEG_10_ has a more inter-connected crystalline morphology via the 2D structure of EEG, as compared to that of the pristine P3HT, which improves the connectivity among the grain boundaries of the P3HT domains. The active channel layer composed of only long polymer chains without EEG sheets has smaller domains, making charge carrier transfer in the active layer difficult. This can be overcome by incorporating highly conductive and large-area EEG, which acts as both helper for enhancing crystallinity and conducting bridge for charge carrier transfer in the active channel layer [43].

### 3.2. Measurement of Mechanical Properties of P3HT/EEG Nanocomposite Films via Film-on-Elastomer Methods

In general, the mechanical properties of organic thin films used as active channel layers are the main factors that determine the flexibility and durability of the organic devices. However, it is difficult to measure the mechanical properties of thin films using conventional characterization methods such as tensile tests. Fortunately, novel methods for measuring the mechanical properties of organic thin films have been developed [44,45]: FOE method is a powerful and accurate technique for quantitatively measuring the mechanical properties of thin films [46,47].

**Figure 6:**
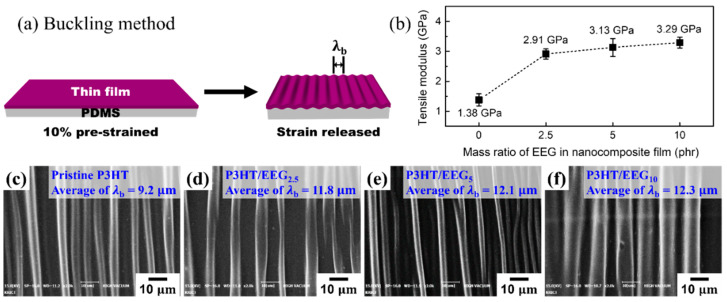
(**a**) Schematic illustration of buckling method. (**b**) Tensile modulus and (**c**–**f**) SEM images of buckling waves on transferred P3HT and the P3HT/EEG nanocomposite films.

The tensile modulus of thin films can be obtained using the FOE-based buckling method. This technique takes advantage of the buckling wave that appears on organic thin films coated on an elastic substrate (Figure 6a). The tensile modulus of the nanocomposite films was calculated by the following equation:

$$E_{f}=3E_{s}(\frac{1-{\nu_{f}}^{2}}{1-{\nu_{s}}^{2}}){\left(\frac{\lambda_{b}}{2\pi d_{f}}\right)}^{3}$$

where *E*_f_ is the tensile modulus of the film, *E*_s_ is the tensile modulus of the PDMS substrate (0.9–1.0 MPa), $\nu_{f}$ is the Poisson’s ratio of the film (0.35), $\nu_{s}$ is the Poisson’s ratio of the PDMS substrate (0.5), and *d*_f_ is the film thickness (200 nm) [30,48]. The buckling wavelength ($\lambda_{b}$) was measured experimentally. The SEM images showed that the buckling wavelengths increased (Figure 6c–f) as the EEG content of the nanocomposite films increased. Compared with the transferred P3HT film, the tensile modulus of the P3HT/EEG nanocomposite films also tended to increase as the mass ratio of EEG increased (Figure 6b). The P3HT/EEG_10_ nanocomposite film exhibited approximately 2.4 times higher modulus (3.29 GPa) than that of the P3HT film (1.38 GPa). Nanocomposite films with higher EEG contents (~10 phr) have a highly crystalline structure, where the P3HT and EEG are tightly held together compared to an amorphous structure [48,49]. However, organic thin films with even higher EEG contents (~20 phr) are too brittle and stiff to form the buckling structure due to the excessive aggregation of EEG sheets. It was confirmed the modulus and stiffness of nanocomposite films were strongly affected by the mass ratio of EEG in the films.

**Figure 7:**
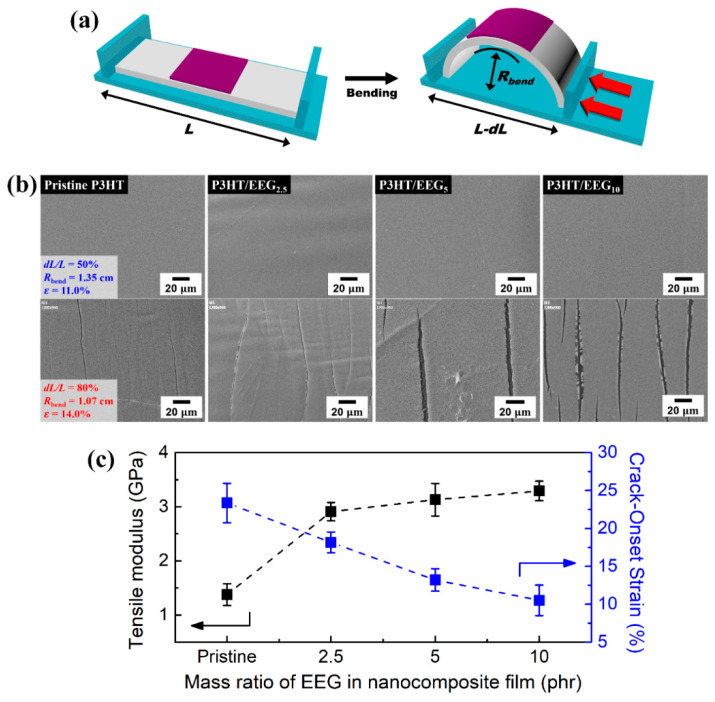
(**a**) Schematic illustration of bending test and (**b**) SEM images of the P3HT and P3HT/EEG nanocomposite films at bending state of *dL/L* = 50% (*R* = 1.35 cm, ε = 11.0%) and *dL/L* = 80% (*R* = 1.07 cm, ε = 14.0%). (**c**) Comparison between tensile modulus and crack-onset strain depending on the mass ratio of EEG in nanocomposite films.

The bending properties (flexibility) of the nanocomposite films were also characterized using the FOE method. The FOE method can provide the effective strain on thin films at a certain bending radius. The measurements were performed for nanocomposite films on the rectangular PDMS substrate with initial length of 60 mm. The transferred P3HT and nanocomposite films on PDMS were bent when the lengths of the PDMS rectangles were reduced by compressive force (Figure 7a). The bending radii of the films were calculated by the following equation:

$$R_{\mathrm{bend}}=\frac{L}{2\pi \sqrt{\frac{dL}{L}-\frac{\pi^{2}h_{s}^{2}}{12L^{2}}}}$$

where *R*_bend_ is bending radius, *L* is the initial length of the PDMS substrate, *dL* is the change in length, and *h*_s_ is the substrate thickness. The effective bending strain (ε) is defined as *h*_s_/(2*R*_bend_) [42,50].

The flexibility and stretchability of the films are closely related to the crack-onset strain, the minimal strain level at which microcarcks begin to develop. Figure 7b shows the SEM images of the transferred P3HT and P3HT/EEG nanocomposite films at bending state of *dL/L* = 50 and 80%. At *dL*/*L* = 50%, no cracks were observed on the surface of both P3HT and nanocomposite films, where the bending radius and strain were 1.35 cm and 11%, respectively. From this observation, it is confirmed that the P3HT/EEG nanocomposite films have good bending flexibility over 10.0% of bending strain in the wide range of the EEG content (from 0 to10 phr). However, cracks appeared on the P3HT/EEG_5_ and P3HT/EEG_10_ nanocomposite films when *dL*/*L* reached approximately 80% (*R*_bend_ = 1.07 cm, ε = 14.0%). The transferred P3HT and P3HT/EEG_2.5_ nanocomposite film only showed wrinkles and microcracks on the surface at bending state of *dL/L* = 80%.

Figure 7c shows the crack-onset strain as a function of mass ratio of EEG in nanocomposite films. To clarify the correlation between the bending flexibility and tensile modulus of nanocomposite films, tensile modulus data were added and compared to the crack-onset strain. It was found that these two properties have an opposite tendency; the tensile modulus increased and bending flexibilities decreased when the mass ratio of the EEG in the films increased. The P3HT/EEG_10_ nanocomposite film exhibited lower crack-onset strain (11.5 %) than the others, which means that P3HT/EEG_10_ film has higher tensile modulus (3.29 GPa) but lower bending flexibility. From the comparison between the tensile modulus and crack-onset strain, it was concluded that the P3HT/EEG nanocomposite films have superior mechanical properties to that of pure P3HT film in the wide range of the EEG content (from 0 to 10 phr) while maintaining the good bending flexibility over 10.0% of bending strain (bending radius of 1.5 cm).

**Figure 8:**
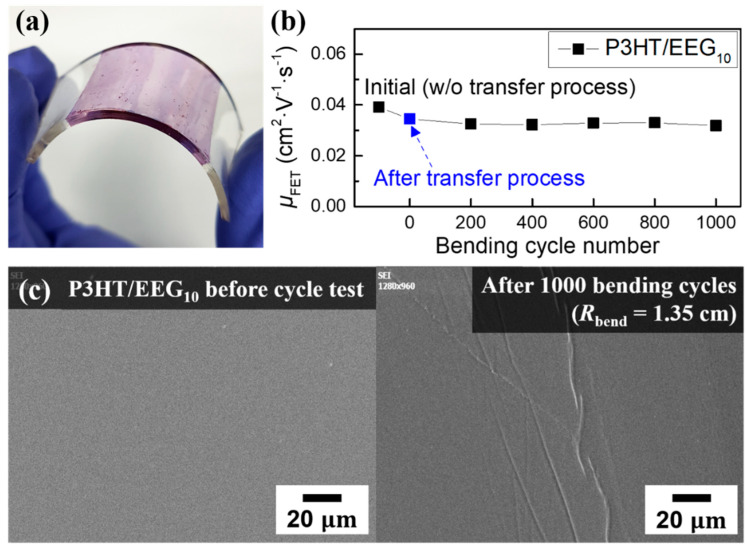
(**a**) Photo image of the P3HT/EEG_10_ nanocomposite film with the Poly(dimethylsiloxane) (PDMS)/PEDOT:PSS substrate under bending condition (*R*_bend_ = 1.35 cm). (**b**) Change of the field-effect mobility depending on the bending cycles. (**c**) SEM images of the P3HT/EEG_10_ nanocomposite film before (left) and after (right) cyclic bending test.

To evaluate the bending durability of the nanocomposite films, we carried out a cyclic bending test (*R*_bend_~1.35 cm, bending strain ~11%) for the P3HT/EEG_10_ nanocomposite film, which showed the highest field-effect mobility and tensile modulus. P3HT/EEG_10_ nanocomposite films were transferred onto the flexible PDMS/PEDOT:PSS substrates by solution floating method for the cyclic bending tests (Figure 8a). After 0, 200, 400, 600, 800, and 1000 bending cycles, each sample was transferred to the Si/SiO_2_ substrate with Au S/D electrodes. Figure 8b shows the change of the field-effect mobility depending on the bending cycles. Difference between the data points of ‘initial’ and ‘0 cycle’ is without and with the transfer process of the nanocomposite film from the flexible PDMS/PEDOT:PSS substrate to Si/SiO_2_ substrate, respectively. The field-effect mobility was slightly decreased from 0.0391 to 0.0345 cm^2^V^−1^s^−1^ from the points of initial to 0 cycle (Figure 8b and Figure S3, Supporting Information). Such a slight degradation of the device performance before the cyclic bending test may be caused by the introduction of small defects on the nanocomposite film during the multiple-transfer processes (floating on the water surface ε → PDMS/PEDOT:PSS substrate → Si/SiO_2_ substrate) [51]. After 0 cycles, however, the device performance stabilized, and the mobility values remained nearly unchanged until the number of bending cycles reached 1000; average mobility of the OFETs based on the P3HT/EEG_10_ nanocomposite films was 0.325 cm^2^V^−1^s^−1^ after the cyclic bending test. Although some wrinkles were founded on the P3HT/EEG_10_ nanocomposite film, there was no crack on the film surface after 1000 cycles of bending test (Figure 8c). We confirmed that the P3HT/EEG_10_ nanocomposite film endured 1000 bending cycles and demonstrated the stable device performance during cyclic bending tests.

It has been reported that a semiconducting polymer film has both bending and stretching flexibility to some extent, and we already mentioned above that the composite film has less pliability than pristine P3HT film. Savagatrup et al. reported that the P3HT:PCBM blend film had lower flexibility because the blend film showed almost 70% lower crack-onset strain than that of the pristine P3HT film [30]. In addition, Kim et al. also reported that the conjugated polymer:PCBM blend films have poorer flexibility and durability than that of the pure polymer semiconductor films under mechanical bending situation [52]; solar cell properties of the organic devices based on the conjugated polymer:PCBM blend films were degraded at only 150 cycles of bending test because of the crack propagation in the blend film by the mechanical deformation. However, in the case of P3HT/EEG nanocomposite film, the highly conductive and large-area EEG sheets act as not only an additive for improving the electrical properties of the OFET devices but also a structural support in the composite film because the 2D flat structure of EEG has more morphological advantages than the spherical shape of PCBM. Therefore, the P3HT/EEG_10_ nanocomposite films can tolerate the durability test under 1000 cycles of mechanical bending.

From the FOE results, we confirmed that the tensile modulus of P3HT/EEG nanocomposite films increased since the nanocomposite with higher EEG contents (~10 phr) have a highly crystalline structure, where the P3HT and EEG are tightly held together compared to an amorphous structure. Moreover, the P3HT/EEG_10_ nanocomposite films, which showed the highest field-effect mobility, maintained good bending flexibility and durability for the potential applications as active layers in flexible organic devices.

## 4. Conclusions

We prepared and characterized the P3HT/EEG nanocomposite films with various EEG contents for use as active channel layers in OFET devices. The OFET fabricated with the P3HT/EEG_10_ nanocomposite film exhibited a nearly twice higher field-effect mobility of 0.0391 cm^2^·V^−1^·s^−1^ compared with the OFET based on the pristine P3HT (0.027 cm^2^·V^−1^·s^−1^), and showed one order of magnitude amplification of the OFET on/off ratio (~10^4^). The crystallinity and mechanical properties of the nanocomposite films were also highly dependent on the mass ratio of EEG; as the EEG content increased from 0 to 10 phr, the crystallinity of the P3HT/EEG nanocomposite film increased. Moreover, from the FOE results, it was confirmed that the P3HT/EEG_10_ nanocomposite film exhibited approximately 2.4 times higher modulus (3.29 GPa) than that of the P3HT film (1.38 GPa), while maintaining the good bending flexibility and durability over 10.0% of bending strain (bending radius of 1.5 cm) and bending cycles (1000 cycles). Based on these results, we concluded that the P3HT/EEG nanocomposite films deposited onto both rigid and flexible substrates using the solution floating method can be an excellent active channel material for high performance and flexible organic devices.

## Figures and Tables

**Table 1:** Device performance parameters of OFETs based on pristine P3HT and P3HT/EEG nanocomposite films.

Parameters	Pristine P3HT	P3HT/EEG_2.5_	P3HT/EEG_5_	P3HT/EEG_10_	P3HT/EEG_20_
***μ*_FET_ (cm^2^·V^−1^·s^−1^)**	0.0227	0.0223	0.0278	0.0391	−
***I*_on_/*I*_off_**	~10^3^	~10^3^	~10^4^	~10^4^	−
***V*_Th_ (V)**	−6.97	−8.87	−11.8	−15.8	−

## Supplementary Materials

The following are available online at https://www.mdpi.com/2073-4360/12/5/1046/s1, Figure S1: Photo images of the bended sample at fixed *dL/L* = 50% in the SEM chamber, Figure S2. Optical image of aggregation of P3HT/EEG_20_ nanocomposite solution, Figure S3: Transfer characteristics of OFET devices based on the P3HT/EEG_10_ nanocomposite film at 0 (after transfer process), 200, 400, 600, 800 and 1000 cycles of bending test.
