# Supplementary material for: Preparation of Nanocomposite-Based High Performance Organic Field Effect Transistor via Solution Floating Method and Mechanical Property Evaluation
Source: Polymers (Basel). 2020 May 2;12(5):1046. doi: 10.3390/polym12051046 (PMC7284566; doi:10.3390/polym12051046)
Supplement: Supplementary file 1 [file polymers-12-01046-s001.pdf]

polymers

MDPI

Supporting Information

Preparation of Nanocomposite-based High Performance Organic Field Effect Transistor via Solution Floating Method and Mechanical Property Evaluation

Youn Kim [1,2], Yeon Ju Kwon [1], Seungwan Ryu [1], Cheol Jin Lee [2,*] and Jea Uk Lee [1,*]

[1] Carbon Frontier Research Center, Korea Research Institute of Chemical Technology (KRICT), Daejeon 34114, Korea; younkim@krict.re.kr (Y.K.); kyj0905@krict.re.kr (Y.J.K.); skyzoop@krict.re.kr (S.R.)
[2] School of Electrical engineering, Korea University, Seoul 02841, Korea
* Correspondence: cjlee@korea.ac.kr (C.J.L.); leeju@krict.re.kr (J.U.L.); Tel.: +82-42-860-7392 (J.U.L)

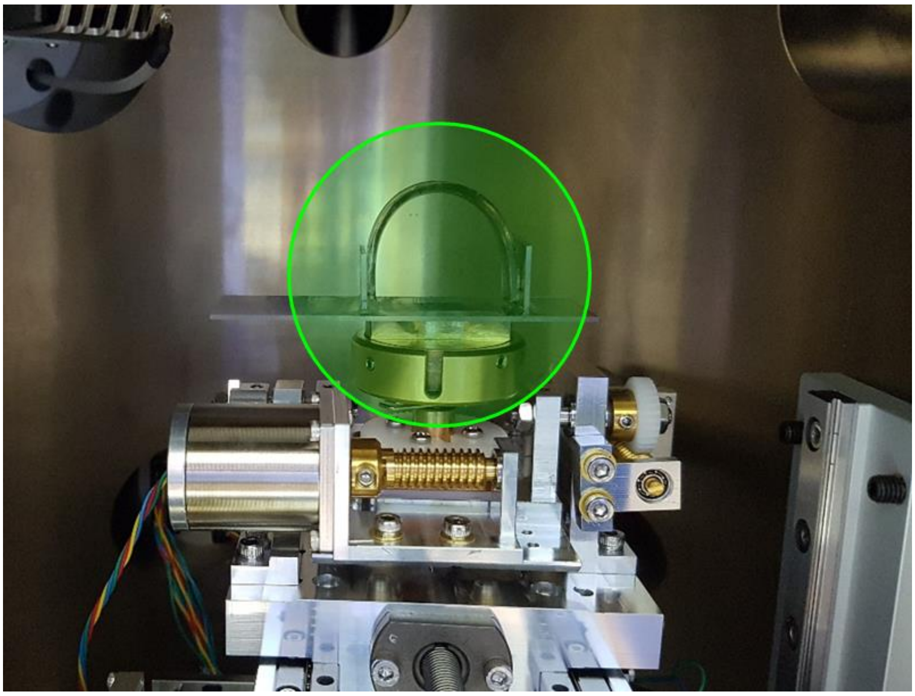

Figure S1. Photo images of the bended sample at fixed dL/L = 50% in the SEM chamber.

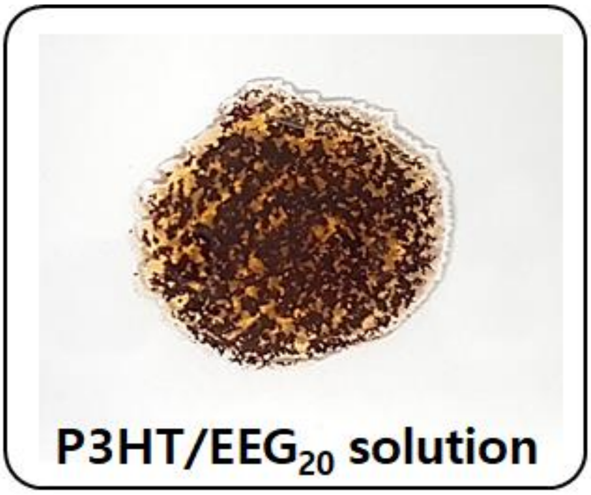

Figure S2. Optical image of aggregation of P3HT/EEG_{20} nanocomposite solution.

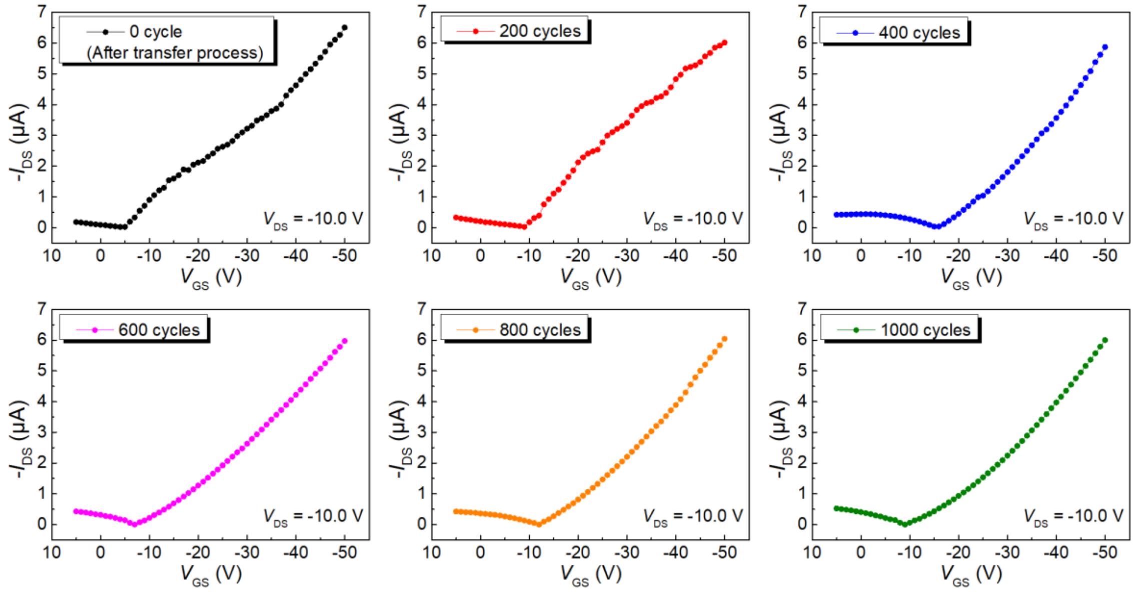

Figure S3. Transfer characteristics of OFET devices based on the P3HT/EEG_{10} nanocomposite film at 0 (after transfer process), 200, 400, 600, 800 and 1000 cycles of bending test.
